# Clinical Perspectives of Gut Microbiota in Patients with Chronic Kidney Disease and End-Stage Kidney Disease: Where Do We Stand?

**DOI:** 10.3390/biomedicines11092480

**Published:** 2023-09-07

**Authors:** Alexandru Cosmin Pantazi, Mustafa Ali Kassim Kassim, Wassan Nori, Liliana Ana Tuta, Cristina Maria Mihai, Tatiana Chisnoiu, Adriana Luminita Balasa, Larisia Mihai, Ancuta Lupu, Corina Elena Frecus, Vasile Valeriu Lupu, Sergiu Ioachim Chirila, Anca Gabriela Badescu, Laurentiu-Tony Hangan, Simona Claudia Cambrea

**Affiliations:** 1 Faculty of Medicine, “Ovidius” University of Constanta, 900470 Constanta, Romaniatuta.liliana@univ-ovidius.ro (L.A.T.);; 2 College of Medicine, Mustansiriyah University, Baghdad 10052, Iraq; dr.wassan76@uomustansiriyah.edu.iq; 3 Clinical Emergency Hospital of Constanta, 900591 Constanta, Romania; 4 Faculty of Medicine, “Grigore T. Popa” University of Medicine and Pharmacy, 700115 Iasi, Romania; valeriulupu@yahoo.com

**Keywords:** gut microbiota, kidney disease, dysbiosis, probiotic, uremic toxin

## Abstract

The gut microbiota (GM) plays a vital role in human health, with increasing evidence linking its imbalance to chronic kidney disease and end-stage kidney disease. Although the exact methods underlying kidney-GM crosstalk are not fully understood, interventions targeting GM were made and lay in three aspects: diagnostic, predictive, and therapeutic interventions. While these interventions show promising results in reducing uremic toxins and inflammation, challenges remain in the form of patient-specific GM variability, potential side effects, and safety concerns. Our understanding of GMs role in kidney disease is still evolving, necessitating further research to elucidate the causal relationship and mechanistic interactions. Personalized interventions focusing on specific GM signatures could enhance patient outcomes. However, comprehensive clinical trials are needed to validate these approaches’ safety, efficacy, and feasibility.

## 1. Introduction

Microbes colonize every surface of the human body, but an increasing proportion of microbes inhabit the intestine. Consequently, gut microbiota (GM) is regarded as a “forgotten organ”. In a healthy state, GM plays several critical roles in our bodies, such as helping to metabolize nutrients, preserving the structural soundness of the gut’s mucosal barrier, moderating immune responses, and providing defense against harmful pathogens [1,2]. A microbiota describes all microorganisms that colonize the epidermis, respiratory tract, genital system, and especially the gastrointestinal tract. GM is constantly evolving and displaying a wide diversity within the same person and in comparison, to others [3]. GM connects with vital organs, including the brain, bone marrow, cardiovascular system (CVS), kidney, body’s immune system, and the central nervous system, and has been seen as a potential cause for a variety of diseases in the aforementioned organs [4,5,6,7,8,9,10]. GM activates immune cells derived from bone marrow, resulting in a low-grade inflammatory reaction that affects the brain and kidneys via circulation [11]. Simultaneously, peripheral stimuli affect the brain and modulate neural inputs to the kidney, intestine, and lymphoid organs [11]. This bidirectional relationship lends credence to the notion that GM modulation is an innovative method for the management of kidney diseases [12,13].

Dysbiosis is an imbalance or perturbation in the GMs composition that results in a proliferation of harmful bacteria like *Enterobacteriaceae* or a reduction in beneficial bacteria like *Bifidobacterium* and *Lactobacillus* [14]. For individuals dealing with ongoing kidney conditions, such as chronic kidney disease (CKD) and terminal kidney failure, often referred to as end-stage kidney disease (ESKD), the harmonious and mutually advantageous connection is disrupted, leading to an imbalance known as dysbiosis [15]. The consequences of this dysbiosis go beyond the gut and impact the kidneys via the so-called gut–kidney axis [13]. One of these adverse outcomes is the overproduction of uremic toxins such as indoxyl sulfate and p-cresyl sulfate, which are derived from bacterial metabolism of dietary amino acids [16]. In healthy individuals, these toxins are efficiently excreted by the kidneys, but in CKD and ESKD, their clearance is significantly reduced, leading to a high plasma concentration of these toxins [17]. Increasing evidence confirms that dysbiosis by itself contributes to CKD development and progression [18].

CKD and ESKD affect roughly 10 percent of the world’s population and impose a substantial financial burden on the healthcare system [19]. Owing to an insufficient understanding of both the origin and the bodily responses associated with CKD, there have not been any significant advances in decades, despite efforts to slow the progression of CKD [20,21].

Recently, interest in modulating GM has increased; the kidney–GM bidirectional relationship has emerged as a novel modulator for kidney diseases [22]. A growing body of research has recently been directed toward GMs role in forecasting and improving health [23]. Though GMs role in CKD and ESKD was researched, the clinical perspective of GM application in those specific patients was not well examined. Here, we discuss the kidney-GM interplay and how this bidirectional relationship can be appreciated in practice in diagnosing and preventing CKD-related morbidities. Moreover, therapeutic avenues for modulating GM were evaluated along with their safety profile.

## 2. The Kidney GM Crosstalk

A. *How do CKD and ESKD contribute to disturbed GM?*

**Figure 1:**
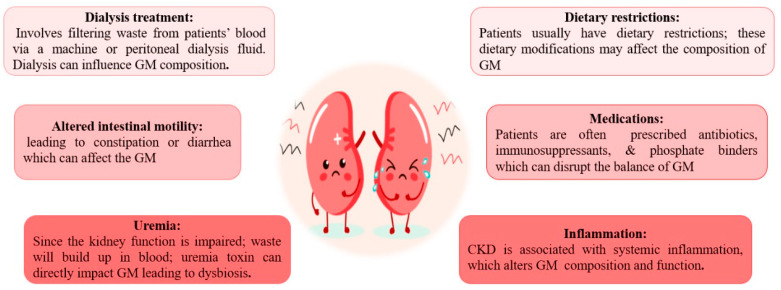
Causes of altered gut microbiota in patients with chronic kidney disease and end-stage kidney disease.

CKD cases have common dietary restrictions, like low protein intake and avoiding foods rich in potassium and phosphorus [24,25,26], which affect the composition of GM [27]. Building-up of waste products (uremia toxin) in the blood owing to impaired kidney function directly affects the GM and leads to dysbiosis [28]. Prescribed Medications: Patients are often on antibiotics, immunosuppressants, and phosphate binders [29,30]. Collectively, they can disrupt the balance of GM [31]. Patients with ESKD often require dialysis, which involves filtering waste products from the bloodstream via a machine or peritoneal dialysis fluid [32]. Dialysis by itself can impact GM composition [33]. A state of systemic inflammation associated with CKD and ESKD may alter the GM composition and function [34]. Chronic kidney disease patients often suffer altered intestinal motility, leading to constipation or diarrhea. These bowel changes can impact the GM [35] (Figure 1).

B. *How does disturbed GM impact CKD and ESKD Progression?*

It is important to note that these causes may interact with each other, leading to a complex interplay between gut dysbiosis and chronic renal disease progression. As renal function declines, the capacity to eliminate toxins decreases, leading to a detrimental cycle of gut dysbiosis and exacerbating uremia [36]. Reduced microbial diversity has been linked to an increase in disease severity and deteriorating health outcomes [37]. Some GM can transform specific toxins into perilous byproducts, which intensify renal damage and induce widespread inflammation within the body [38]. GM plays a pivotal role in the processes of nutrient metabolism and energy extraction [39]. However, when dysbiosis occurs, it can have detrimental effects on nutrient assimilation and metabolism, leading to conditions such as malnutrition or an imbalanced energy equilibrium [40]. The presence of altered gut microbiota leads to the disruption of the intestinal barrier function, which permits the passage of microbial components and harmful substances into the bloodstream [41]. This, commonly referred to as “Leaky gut syndrome” or “endotoxemia”, subsequently initiates a systemic inflammatory response [42]. Dysbiosis and the associated modification of GM can result in an impaired immune response, making the host more susceptible to infections and inflammatory diseases [43,44].

C. *How does disturbed GM contribute to CKD- and ESKD-related complications?*

There is growing data indicating a connection between dysbiosis and complications associated with CKD, including high blood pressure, cardiovascular incidents, disorders related to minerals and bones (MBD), and cognitive impairments.

❖ *CKD- and ESKD-related cardiovascular disease*

Several studies find that diverse mechanisms play a role in the development and progression of cardiovascular disease, a major mortality cause among those patients [45,46]. These include increased reactive oxygen species (ROS) production, leukocyte activation, pro-inflammatory cytokines production, myocyte hypertrophy, and dyslipidemia. This relationship between the digestive tract and the heart is known as the gut–heart axis [47,48]. Lin et al. [49] found an association between elevated pCS levels and increased CVS mortality in CKD patients. Conversely, low TMAO was associated with a 1.7-fold greater risk of severe CVS events [50].

❖ *Cognitive psychiatric disorders*

Cognitive psychiatric disorders are prevalent among CKD patients and are associated with an increase in morbidity and mortality [51]. The gut–brain axis promotes dysregulation of the hypothalamus–pit axis [52]. The contribution of gut-microbiota-derived toxins to cognitive dysfunction is conveyed through mechanisms like direct toxicity or other potential influences, such as oxidative stress, inflammation, dysfunction of endothelial cells, and vascular calcification [53]. Lin et al. [54] demonstrated in a study involving 260 hemodialytic cases that the circulating free form of IS is substantially associated with decreased cognitive function, especially in the memory domain, mental manipulation, and language ability.

❖ *CKD—disorder of bone and minerals*

This syndrome was recently renamed to encompass biochemical, skeletal, and CVS pathogenesis in addition to bone disease [55]. It was suggested that elevated GM-derived toxins contribute to the onset of bone abnormalities in CKD [56]. Previous research has shown that increased levels of IS can impede the function of osteoblasts and have a restraining effect on osteoclasts and parathyroid hormone, which may consequently affect the bone remodeling process in patients with CKD [57,58].

D. *How does disturbed GM affect the production of key metabolic intermediates such as short-chain fatty acids?*

Multifaceted interactions characterize the relationship between GM and the health of individuals with CKD. Entities such as GM are responsible for the production of key metabolic intermediates, such as short-chain fatty acids (SCFAs), via the process of fermenting dietary fiber [59,60]. Compromised renal function has the potential to disturb the equilibrium of these entities and metabolic pathways, thereby potentially exacerbating CKD and disease progression [59,60]. SCFAs were intimately linked to diverse physiological processes, such as immune function, inflammation, and metabolism [59]. SCFAs are a class of organic compounds with short carbon chains (2 to 6 carbons, typically). The intestinal GM produces them along with other complex carbohydrates [59].

The principal SCFAs synthesized are acetate, propionate, and butyrate. SCFAs role has been extensively investigated in patients with CKD and may be summarized as energy metabolism, modulating immunity, maintaining gut integrity, and CVS wellbeing [61]. 

First, SCFAs once absorbed into the circulation act as a host’s energy source. They are, presumably, influencing insulin sensitivity and weight management through their effect on glucose and lipid metabolism [62]. Second, SCFAs stimulate the production of regulatory T cells (Tregs) and other immune cells that assist in maintaining immune homeostasis and reducing excessive inflammation [63]. Thus, SCFAs modulate immunity and affect the equilibrium between pro-inflammatory and anti-inflammatory responses [63]. For that, reduced SCFA production tends to impair the immune system, amplify inflammation, impair immunological function, and contribute to the advancement of chronic kidney disease (CKD) [63]. Third, butyrate was shown to improve the intestinal barrier’s integrity [64]. It stimulates the production of mucins and tight junction proteins, which are crucial for maintaining the gut barrier integrity. This effect is vital in avoiding the translocation of toxins and bacterial products into the circulation, thereby reducing systemic inflammation [64]. Fourth, SCFAs have been linked with cardiovascular health [65]. They affect blood pressure regulation, lipid metabolism, and endothelial function [65]. All of these are relevant factors in CKD patients, who suffer from an increased risk of cardiovascular complications and form a significant cause of mortality [65].

The impact of short-chain fatty acids within the setting of chronic kidney disease is intricate and diverse. It is essential to note that this relationship is still the subject of active research, and the precise mechanisms by which SCFAs influence CKD have not been fully elucidated [66]. In addition, interventions targeting the intestinal microbiota and SCFA production are being investigated as potential therapeutic strategies for managing the progression of CKD; however, additional research is warranted to establish their efficacy, safety, and possibly lead to innovative methods for treating CKD and its complications.

## 3. What Are the Clinical Applications for Implementing GM in Patients with CKD and ESKD?

**Figure 2:**
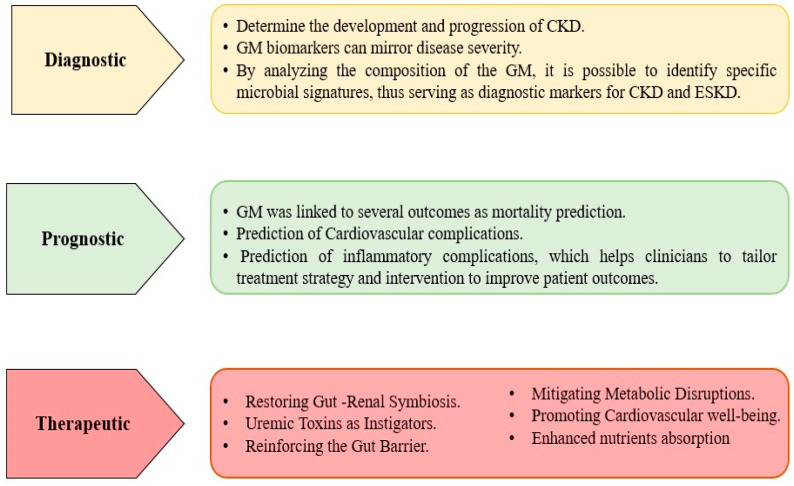
The clinical insight of GM in cases with CKD and ESKD.

The understanding and exploration of GM have paved the way for numerous clinical applications in the management of CKD and ESKD. These applications extend to diagnostic, prognostic, and therapeutic domains (Figure 2).

A. *Diagnostic Applications:*

Investigation of the GMs composition and functionality offers valuable diagnostic insights. In individuals with CKD and ESKD, GM shows a reduction in advantageous bacteria like *Bifidobacterium* and *Lactobacillus*, along with a surge in pathogenic species, including *Enterobacteriaceae* and *Clostridium* [67]. Moreover, the generation of excessive nephrotoxins by dysbiotic GM may determine the development and progression of CKD. Additionally, GM biomarkers can mirror disease severity [68].

The serum levels of two microbiota-derived nephrotoxins, pCS and IS, were significantly linked with GM biomarkers, which suggests a link of gut-metabolite–kidney axis as an etiological factor in renal impairment and confirms their utility as an early diagnostic and prognostic biomarker in CKD [53,69].

Bacterial genes involved in aromatic amino acid metabolism were different across the stages of CKD. For instance, *Escherichia Shigella* spp. (ES spp.) predominates CKD patients’ urine and feces [70,71].

The overrepresented ES spp. was strongly linked to IS levels and was associated with a deteriorating kidney function. Among cases with early stage kidney decline, microbes belonging to the *Ruminococcaceae* family were associated with IS and pCS [72]. *Escherichia coli* (EC) was recognized as an advanced CKD cases biomarker and discriminated cases vs. controls [72]. EC can convert tryptophan into indole [73], which opens a therapeutic avenue added to the diagnostic role via genetic manipulation aiming to reduce indole and IS levels [74]. Despite these appealing results, further clinical trials should be warranted to demonstrate the reduction in IS and pCS through manipulation of GM since the current research was hindered by small sampling and inconclusive results [75].

B. *Prognostic Application:*

The prognostic applications for GM in CKD and ESKD are a burgeoning research field with enormous potential. Those can be grouped into three aspects: mortality prediction, prediction of cardiovascular, and inflammatory complications.

*Mortality Prediction:*

Recent work identified specific GM compositions linked to higher mortality risk among ESKD, such as *Enterococcus* [76]. Moreover, a lower GM diversity was associated with poor outcomes [37]. By advanced sequencing techniques, we may identify GM composition and activity. Hopefully, it will help in establishing a prediction model for high-risk cases [77].

*Cardiovascular Complications:*

Hypertension, atherosclerosis, and heart failure are significant contributors to morbidity and mortality in ESKD [78]. Certain microbial metabolites produced by GM can have direct effects on CVS by interfering with blood pressure regulation or lipid absorption and metabolism [79]. These associations play a prognostic and therapeutic function in preventing or treating CVS challenges [47,80].

*Inflammatory Complications:*

Inflammation is a prevalent manifestation in renal failure patients and plays a role in disease progression and complications [81]. Changes in gastrointestinal permeability caused by dysbiosis lead to the translocation of bacterial products into the blood, resulting in a systemic inflammatory state [82]. Furthermore, the immunomodulatory effect of GM affects cytokine production. Understanding the contribution of GM to inflammatory consequences in CKD patients is a promising strategy for predicting and managing these complications [83]. Despite these encouraging results, additional research is required to validate these findings and develop clinically applicable, robust predictive models.

C. *Therapeutic Applications:*

*Restoring Gut–Renal Symbiosis*: GM holds a key role in our comprehensive health, including renal well-being. Disturbances of this delicate ecosystem trigger a series of adverse events that fuel the progression of CKD and ESKD [84,85,86]. Thus, targeted modulation GM could potentially provide a means to restore renal function by damping inflammation and reducing oxidative stress.

*Uremic Toxins as Instigators*: CKD and ESKD patients suffer a marked buildup of uremic toxins which cannot be eliminated by a diseased kidney. Losing the harmony of GM creates a perpetuating cycle of renal damage [87]. By harnessing the power of specific microbial agents or beneficial bacteria, it might be possible to facilitate the removal of these uremic toxins via alternative routes.

*Reinforcing the Gut Barrier:* the “leaky gut” phenomenon, where increased permeability of the gut wall induces alterations in GM, thereby fostering inflammation [88]. GM modulation strategies have the potential to reinforce gut barrier integrity. By cultivating a balanced microbiota, it is possible to create an environment that minimizes toxin leakage and inflammatory responses, thereby improving renal function.

*Mitigating Metabolic Disruptions*: The presence of CKD is frequently accompanied by metabolic aberrations, including dyslipidemia, insulin resistance, and glucose metabolism irregularities. The GM wields substantial influence on the host metabolism, including aspects such as the production of short-chain fatty acids and the metabolism of bile acids, which has been linked to systemic metabolic health [89]. Efforts to recalibrate the gut microbiota composition might represent an effective strategy to ameliorate these metabolic dysfunctions associated with CKD.

*Promoting Cardiovascular Well-being*: GMs role in promoting CVS health has been increasingly recognized [90,91]. Consequently, the modulation of the gut microbiome might provide substantial benefits in regulating blood pressure and lipid metabolism and reducing vascular inflammation [92]

*Enhanced nutrient absorption*: patients often suffer from malnutrition caused by impaired absorption. GM modulation may improve the absorption of vitamins and minerals, leading to improved nutritional status [93].

*GM can mitigate drug-induced liver injury* (DILI) *and alcoholic liver disease*: GM significantly influences drug metabolism and elimination in CKD and renal disease patients [94]. The capacity of GM to metabolize and modulate drug absorption and distribution contributes to protecting the liver from drug-induced damage [95]. GMs beneficial effect is mediated via multiple pathways: (1) enzymes can metabolize drugs, altering their chemical structure and reducing their toxicity in a process known as biotransformation [96,97], (2) by modulating the body’s immunity [98], and (3) maintaining gut barrier integrity can further protect against hepatic damage [99]. Manipulation of GM can have a potential therapeutic avenue to mitigate hepatotoxicity in CKD patients. GMs beneficial effect in reducing liver toxicity is also seen in alcoholic liver disease (ALD). Where GM interferes with alcohol metabolism, modulates gut permeability, regulates bile acid metabolism, and modulates the immune responses [100]. These interactions can offer a therapeutic target for preventing the progression of ALD [101]. The same effect was noticed in patients with gastrointestinal malignancy, where modulating GM was proposed to reduce cytotoxic drugs’ adverse effects [102].

### Methods by Which GM Balance Is Restored in CKD and ESKD

**Figure 3:**
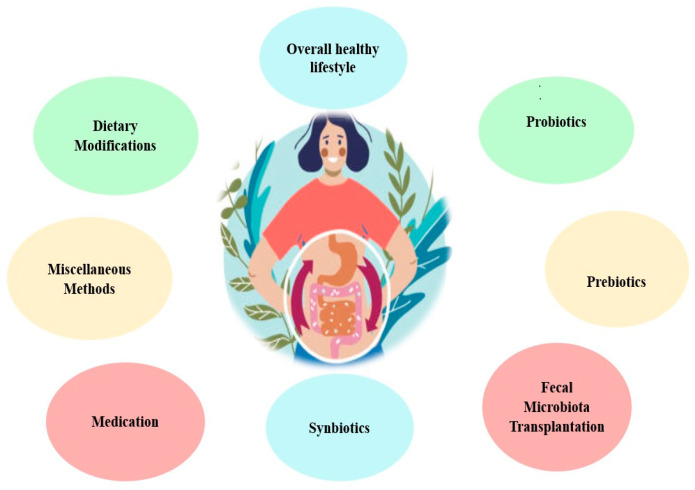
Methods by which GM balance is restored in CKD and ESKD.

Methods for restoring GM have recently emerged as a novel approach for treating many diseases among patients with CKD and ESKD. Many methods exist, and they are summarized in Figure 3.

❖ *Maintaining an overall healthy lifestyle* can positively enhance health. Regular physical activity, techniques for managing tension, enough sleep, and avoiding smoking and excess alcohol consumption may all contribute to a healthier digestive environment [103].
❖ *Dietary modifications:* a personalized dietary plan is often made for CKD and ESKD patients. They are already on low protein intake, limited phosphorus, potassium-rich food, and fluid intake aiming to reduce uremic toxin precursor [104,105]. These modifications can indirectly impact GM [104,106]. Another dietary intervention is the high-fiber diet aimed to improve the reno-protective precursors [107].

Krishnamurthy et al. [108] study (that included 14,543 participants) revealed a notable association between a high-fiber diet, reduced inflammation, and decreased all-cause mortality. However, in the later stages of CKD, diets rich in fiber may possess certain drawbacks, primarily due to the presence of elements like potassium and phosphorus. As a result, it is crucial to offer practical cooking techniques and guidance to ensure their safety. Furthermore, the consumption of foods rich in choline and L-carnitine, which serve as precursors to trimethylamine-N-oxide, such as egg yolk, kidney, liver, meat, and milk, has been found to correlate with a significant buildup of uremic toxins and a decline in the glomerular filtration rate [73].

A new dietary modulation therapy to regulate GM is resistant starch (RS), a distinct form of carbohydrate that experiences partial digestion by human pancreatic amylases, leaving it incompletely broken down [104]. One notable RS variant, high-amylose maize-resistant starch type 2 (HAM-RS2), is commonly found in starchy food sources, such as potatoes, corn, and bananas [105]. When it enters the large intestine, HAM-RS2 serves as a valuable energy resource for beneficial bacteria, such as *Bifidobacterium* and *Lactobacillus* [109,110].

❖ *Certain medications* prescribed for the management of chronic renal disease can affect GM through multiple pathways, either by altering the composition of the gut microbiota or by eliminating both harmful and good bacteria [111]. Some medications harm the intestinal mucosa and alter the gut microbiome [112]. Additionally, immune suppressors tend to depress the immune response and alter the gut environment [113]. Even though these drugs may affect GM, their benefits for dealing with chronic renal disease typically outweigh their potential adverse effects [114].
❖ *Probiotics* are primarily live bacteria, such as *Bifidobacteria* and *Streptococci* species [111]. Their principal therapeutic action is their ability to recalibrate the GM [115]. This equilibrium is reinstated through various mechanisms, including displacing harmful bacteria, fortifying gut barrier integrity, and adjusting the host’s immune response [116,117,118]. Research on probiotics suggested improved renal function and quality of life in CKD patients [119,120,121,122,123].
❖ *Prebiotics* are indigestible food components that help stimulate the growth of specific bacteria in the colon [124]. Various prebiotics have been found to foster the expansion of advantageous bacterial strains such as *Bifidobacteria* and *Lactobacilli species* [125]. Simultaneously, these prebiotics appear to inhibit the growth of certain other bacterial clusters [125]. Prebiotics resist digestion until they reach the colon, where they’re fermented by native bacteria, producing short-chain fatty acids (SCFAs) [126]. These SCFAs enhance gut health and boost the immune response [81]. Research has shown that certain prebiotics can reduce the serum concentrations of specific uremic toxins in patients undergoing hemodialysis [127,128]. Furthermore, lactulose has been found to improve kidney function in animal models by modifying the gut microbiota, inhibiting the production of uremic toxins, and suppressing tubulointerstitial fibrosis [129,130].
❖ *Synbiotics* are a combination of probiotics and prebiotics, used to potentiate the beneficial effects of probiotics. A study found that introducing synbiotics to patients with CKD lowered uremic toxins, specifically pCS [131]. Additionally, a randomized trial was conducted in 2023, which investigated the effects of synbiotics on non-dialyzed CKD patients [132] and reported that synbiotic regimens fostered the proliferation of beneficial bacteria in the gut [127]. It also notably decreased the serum levels of indoxyl sulfate, improved the glomerular filtration rate indicative of better kidney function, and attenuated inflammation [132]. Apart from minor side effects like increased flatulence, synbiotics were deemed to be a safe and effective therapeutic strategy to curb the levels of uremic toxins and inflammation in CKD patients [132].
❖ *Fecal Microbiota Transplantation (FMT)* is a method that entails transferring fecal bacteria and other microscopic entities from a person in good health to another person [133]. The primary goal of FMT is to replace good bacteria that have been killed or suppressed, often using antibiotics, causing harmful bacteria, particularly *Clostridium difficile*, to overpopulate the colon [134]. The idea stems from the observation that CKD and ESKD patients often have altered GM, with an overgrowth of bacteria that produce uremic toxins, such as indoxyl sulfate and p-cresyl sulfate [135]. It is worth mentioning that modulation of gut microbiota is the principal mechanism in probiotics, prebiotics, synbiotics, and fecal microbiota transplantation. Early animal studies have provided some promising findings for treating CKD [136,137]. These findings suggest that FMT could potentially improve kidney function in patients with CKD and ESKD by reducing the levels of uremic toxins. However, it is important to note that these are preliminary findings, and more research is needed to determine the optimal protocol for FMT, including donor selection, preparation and administration of the fecal material, and long-term safety and efficacy monitoring.
❖ *Miscellaneous Methods* include [138]:
  ▪ Blocking LPS and inflammation via synthetic TLR4 antagonists and lipid A analogs.
  ▪ The absorption of uremic toxins can be facilitated by oral adsorbents, dialyzers based on a carbon matrix; infusions of plasma-binding proteins like albumin, and the use of ibuprofen during dialysis.
  ▪ Modulation of renal transporters via meclofenamate.

To summarize, GM modulation presents an exciting frontier in the management of CKD and ESKD. The choice of method must be personalized based on the patient’s condition, the safety and efficacy of the approach, and the patient’s preferences. More research is needed to optimize these interventions and to better understand their long-term effects.

## 4. Evaluation of GM Modulation, Potential Risks, and Considerations

GM modulation in CKD and ESKD is an area of ongoing research. Limited studies have explored the potential benefits, efficacy, and serious side and safety concerns. Although GM modulation is still considered safe, there are potential contraindications for using them in CKD and ESKD. Some factors to consider are summarized in Table 1. Additionally, modulating GM has been evaluated regarding safety concerns, pros, and cons, summarized in Table 2.

## 5. Applications and Limitation of GM Modulation in CKD and ESKD

The research concerning GM application in practice has rapidly evolved in the last decade, especially in CKD and ESKD; we have summarized the latest meta-analytic studies published in the last years in Table 3. While the body of evidence linking GM dysbiosis to the progression and complications of CKD and ESKD is rapidly expanding, many factors still limit its implementation in practice. The utilization of FMT as a therapeutic intervention for CKD and ESKD remains a nascent field of study, characterized by a dearth of comprehensive clinical trials conducted thus far. The complex nature of the subject makes it challenging to formulate precise guidelines for its utilization [148]. Variability among individuals is another limitation since GM composition is distinct for each person, posing challenges in accurately predicting an individual’s response to treatment [149]. Furthermore, the efficacy of the intervention may exhibit individual variability. The potential adverse effects caused by the new strains of GM, such as bloating, diarrhea, or allergic reactions, is another limiting fact [150]. Finally, the potential risks and safety implications of modified GM have yet to be fully understood and evaluated. Additional investigation is required to evaluate possible hazards, complexities, safety issues, and optimal usage.

## 6. Future Perspective and Further Research

Identification of GM as a potential target in the management of CKD and ESKD continues to encounter several challenges. GMs intrinsic variety and diversity among those populations are frequently overlooked in research [164]. Various factors, such as dietary patterns, pharmaceutical interventions, and the presence of concurrent medical conditions, can potentially impact the diversity of GM [165,166]. It is imperative to incorporate strategies to control relevant factors and address individual variations [166]. Unraveling mechanisms of how dysbiosis contributes to CKD progression and ESKD is another aspect that future research should consider. Moreover, addressing various approaches manipulating GM to enhance kidney health allows the evaluation of their efficacy in restoring a healthy GM equilibrium [167]. The tailored treatment procedures that address the unique GM composition added to the patient’s specific characteristics, showing promising potential to enhance patient outcomes [168].

Dietary modification is another promising intervention that potentially influences GM composition, improving kidney function [169,170]. Finally, using state-of-the-art methodologies like metagenomics, met transcriptomics, and metabolomics to thoroughly examine the GM and their functional behaviors in individuals with CKD and ESKD is a new emerging field of research [171]. Despite significant progress in understanding the importance of GM in those populations, there are still significant gaps in knowledge that require deeper investigation and clarification. It is crucial to give precedence to collect mechanistic information, tailored interventions, and assess the broader implications linked to microbial metabolites, dietary patterns, and pharmacological compounds [172]. Investigating the intricate connections between GM and kidney health necessitates the adoption of a multi-disciplinary methodology, which encompasses the expertise of nephrologists, gastroenterologists, immunologists, and microbiome specialists.

## 7. Conclusions

The management of CKD and ESKD presents significant challenges due to their complex nature and the substantial implications they have on the patient’s quality of life. With the increasing understanding of the gut–kidney axis, the role of the GM in these conditions has come to the fore. There is emerging evidence that GM dysbiosis plays a role in the progression and complications of these renal conditions. Thus, GM modulation using various approaches such as dietary interventions, probiotics, prebiotics, synbiotics, and fecal microbiota transplantation could be potential game-changers in this field. While the preliminary findings of these approaches are promising, the evidence is still nascent, and further research is needed to confirm their efficacy, safety, and feasibility. Issues such as individual variability in GM composition, potential adverse effects, interactions with existing medications, and long-term impacts of GM modulation are critical aspects to be addressed. Additionally, the nuanced understanding of whether GM dysbiosis is a cause or a consequence of renal dysfunction is yet to be fully established.

Ultimately, the promising horizon of GM modulation in CKD and ESKD management underscores the importance of further research. Expanding our understanding of the gut–kidney axis and optimizing these interventions could potentially open new avenues in managing these chronic conditions. This underscores the necessity of multi-disciplinary methods to improve the outcomes for these patients and provides hope for a more holistic and effective approach to kidney disease management.

## Figures and Tables

**Table 1:** Potential Risks and Considerations in Gut Microbiota Modulation.

Potential Contraindications	Proposed Side Effect	References
Compromised immunity state	Introducing new GM may disrupt the delicate gut balance and potentially lead to infections.	Thursby et al. [139]; 2017
Medication interaction	Those patients are often on multiple medications, such as immunosuppressants and antibiotics, which impact GM composition and may interact with any introduced microbial modulation.	Chakraborty et al. [32]; 2016
Fluid and electrolyte imbalance	Altering the GM in cases with these imbalances potentially worsens the condition.	Rapa et al. [140]; 2020
Dialysis consideration	The impact of GM therapies on dialysis efficiency or complications is not well understood.	Tang et al. [141]; 2015

**Table 2:** Evaluation of Methods for Modulating Gut Microbiota: Pros, Cons, and Associated Risks.

Method Modulating GM	Pros	Cons	References
Dietary interventions	Effective, non-invasive, and generally well-tolerated have additional benefits, such as improving cardiovascular health.	They require patient compliance. Can be challenging to implement due to individual food preferences and dietary restrictions.	Kaesler et al. [142]; 2021
Probiotics and/or prebiotics	Safe for most individuals.	Effects can be strain-specific and transient, requiring continuous intake. People with compromised immune systems or who are critically ill may be at higher risk for adverse events related to probiotic use. Some strains may interact with medications. Others cause gastrointestinal symptoms, and in addition to that, the cost can also be a limiting factor for some patients.	Simon et al. [143]; 2021 Doron et al. [144]; 2015 Dore et al. [145]; 2019 Lenoir-Wijnkoop et al. [146]; 2019
Fecal Microbiota Transplantation	Safe when it performed under appropriate medical supervision and with proper screening protocols for donors.	Infection transmission; bacterial or viral. Allergic reactions or adverse events related to the procedure itself.	Wynn et al. [147]; 2023

**Table 3:** Gut Microbiota in CKD Patients: A Compilation of Recent Meta-Analysis and Systematic Reviews.

References	Study Modality Number and Type of Studies Examined	Number of Participants and Their Criteria	Key Findings
Thongprayoon et al. [151]; 2018	Meta-analysis was conducted on five randomized controlled trials (RCT)	161 participants with chronic kidney cases (CKD)	Beneficial effects of probiotics on uremic toxins in CKD patients.
Nguyen et al. [152]; 2021	Systematic Review and Meta-analysis on 23 RCT	931 participants On hemodialysis patients	Supplementation with probiotics, prebiotics, and synbiotics significantly decreased circulating levels of various uremic toxins and inflammatory biomarkers. A potential therapeutic benefit in alleviating uremic toxin levels, oxidative stress, and inflammation in hemodialysis patients.
McFarlane et al. [153]; 2019	Systematic Review and Meta-analysis On 16 RCT	645 participants adults and children with CKD.	Prebiotics supplementations have slightly reduced serum urea concentration. However, the evidence was limited.
Yu et al. [128]; 2022	Network Meta-analysis on 25 RCT	1106 participants in ESKD With Dialysis	Prebiotics were found to be effective in reducing certain inflammatory markers and uremic toxins. Synbiotics were effective in reducing CRP and endotoxin levels. Probiotics were beneficial in alleviating gastrointestinal symptoms. This study provides better clinical decisions in treating ESRD patients.
Takkavatakarn et al. [154]; 2021	Systematic Review and Meta-analysis on 38 articles including observational and RCTs.	2492 participants with CKD on dialysis	Protein-bound uremic toxins, including indoxyl sulfate and p-cresyl sulfate, are linked with increased cardiovascular risks in CKD. Strategies such as prebiotics, synbiotics, and AST-120 effectively reduce these toxins.
Liu et al. [155]; 2022	Systematic Review and Meta-analysis on 23 RCT	842 participants with CKD	Probiotics favorably influenced markers of creatinine, oxidant stress, inflammation, and certain uremic toxins in CKD patients.
Yang et al. [107]; 2021	Meta-analysis on 10 RCT	292 participants With CKD	Dietary fiber supplementation can significantly reduce levels of specific uremic toxins in CKD patients. This provides evidence for the clinical recommendation in practice.
Liu et al. [156]; 2020	Systematic Review and Meta-analysis on 16 RCT	605 participants with CKD	Probiotics significantly decreased serum levels of certain inflammatory cytokines in CKD patients, such as CRP and IL-6. They did not significantly affect serum uremic toxin levels, including creatine, urea, uric acid, PCS, and IS. The results help treatment decisions in clinical practice.
Tao et al. [157]; 2019	Meta-analysis on 10 RCT	359 cases with CKD to assess progression	The study suggests that probiotics can reduce urea levels in non-dialysis CKD patients.
Jia et al. [158]; 2018	Systematic Review and Meta-analysis on 8 RCT	261 CKD patients (stage 3 to 5) with and without dialysis	Dysbiosis of the intestinal microbiota may accelerate CKD progression by increasing urea toxin levels. Probiotics have been recognized to maintain the physiological balance.
Jia et al. [159]; 2021	Systematic Review and Meta-analysis on 5 RCT	179 CKD cases	A significant reduction in blood urea nitrogen, serum creatinine, and interleukin (IL)-6 levels in the RS2 group. The findings suggest that RS2 might improve residual renal function in MHD patients and reduce proinflammatory responses.
Chen et al. [160]; 2023	Meta-analysis on 18 RCT	237 cases on Dialysis	Probiotics, prebiotics, and synbiotics supplements could reduce levels of C-reactive protein, interleukin 6, and indoxyl sulfate and increase high-density lipoprotein cholesterol compared to the control group.
Wang et al. [161]; 2022	Meta-Analysis examined 16 case-control or cross-sectional studies	1022 participants (578 patients with Diabetic KD and 444 Healthy controls)	Patients with diabetic kidney disease (DKD) had significantly decreased bacterial richness. The gut microbiota of patients with DKD had specific features characterized by the expansion of genera like *Escherichia*, *Citrobacter*, and *Klebsiella*, and depletion of *Roseburia*. These microbial taxa might be closely related to DKD and could serve as potential targets for DKD management.
Zheng et al. [162]; 2021	Meta-Analysis examined 13 RCT	671 CKD cases	Microbial therapies significantly reduced levels of C-reactive protein, malondialdehyde, total cholesterol, and low-density lipoprotein cholesterol. Increased glutathione levels, total antioxidant capacity, and high-density lipoprotein cholesterol in CKD patients compared to placebo groups. The findings support the potential use of probiotic, prebiotic, and synbiotic supplements in improving cardiovascular risk factors in CKD patients.
Dai et al. [123]; 2022	Meta-Analysis examined 10 RCT	552 participants with diabetic KD	Probiotics can delay renal function injury, improve glucose and lipid metabolism, and reduce inflammation and oxidative stress in DKD patients.
Li et al. [163]; 2023	Meta-Analysis examined 21 cohort, case-control, nested case-control, or analytic cross-sectional studies	15,637 participants that were non-CKD vs. non-black dialysis patients.	Non-dialysis CKD patients and non-black dialysis patients with the highest circulating TMAO concentration had an increased risk of all-cause mortality. Non-black dialysis patients with the highest TMAO concentration also had an increased risk of cardiovascular mortality. Increased circulating TMAO concentrations are associated with higher mortality risks in specific CKD patient groups.

## Data Availability

No new data were generated.
